# The Diagnostic Accuracy of an Intraoperative Frozen Section Analysis and Imprint Cytology of Sentinel Node Biopsy Specimens from Patients with Uterine Cervical and Endometrial Cancer: a Retrospective Observational Study

**DOI:** 10.1007/s12253-020-00822-2

**Published:** 2020-05-26

**Authors:** Tomohito Tanaka, Shunsuke Miyamoto, Shinichi Terada, Yuhei Kogata, Satoe Fujiwara, Yoshimichi Tanaka, Kohei Taniguchi, Kazumasa Komura, Kazuhiro Yamamoto, Takashi Yamada, Masahide Ohmichi

**Affiliations:** 1 Department of Obstetrics and Gynecology, Osaka Medical College, 2-7, Daigaku-machi, Takatsuki, Osaka 569-8686 Japan; 2 Translational Research Program, Osaka Medical College, Takatsuki, Japan; 3 Radiology, Osaka Medical College, Takatsuki, Japan; 4 Pathology, Osaka Medical College, Takatsuki, Japan

**Keywords:** Endometrial cancer, Cervical cancer, Sentinel node biopsy, Frozen section, Imprint cytology

## Abstract

Sentinel node biopsy (SNB) may be a decision-making tool for function preservation surgery, including radical trachelectomy and ovary preservation in the treatment of cervical and endometrial cancer. The intraoperative diagnosis is important for guiding treatment decisions for patients with these conditions. Three hundred seventy-one patients with cervical and endometrial cancer received SNB with an intraoperative frozen section analysis and imprint cytology. The sentinel node was cut in half, parallel to the longest axis, to obtain the maximum section area. After performing imprint cytology, one half was used to create a frozen section. The specimen was cut at 2-mm intervals into 5-μm-thick sections, which were subjected to hematoxylin and eosin staining. The diagnostic accuracy of intraoperative frozen section analyses and imprint cytology was compared to the final pathological diagnosis. Among 951 detected sentinel nodes, 51 nodes were found to be positive in the final pathological diagnosis. The sensitivity of a frozen section analysis, imprint cytology and the combination of the two modalities was 76.5%, 72.6%, and 92.2%, respectively. The specificity of a frozen section analysis and imprint cytology was 100%. The negative predictive value of a frozen section analysis and imprint cytology was 98.7% and 98.5%, respectively. In these settings, the accuracy of the frozen section analysis and imprint cytology in the evaluation of SNB specimens was considered acceptable; however, the sensitivity of the combined approach was higher in comparison to when a frozen section analysis or imprint cytology was performed alone.

## Introduction

The sentinel lymph node (SLN) is the primary lymph node from which cancer cells spread through the lymph trunk. Cases in which no cancer cells are detected in the SLN are predicted to be free of metastasis at that site. One advantage of sentinel node biopsy (SNB) is that it may reduce the need for systematic lymph node dissection. Moreover, if the SLN status is confirmed during surgery, SNB may be an important decision-making tool for function sparing surgery, including radical trachelectomy for the treatment of cervical cancer and ovary preservation in the treatment of endometrial cancer. Sentinel navigation surgery, including minimally invasive and function-sparing surgery, may be recommended for early-stage uterine cancer. The current study evaluated the diagnostic accuracy of intraoperative frozen section analyses and imprint cytology in the diagnosis of SNB specimens from patients with uterine cancer.

## Materials and Methods

### Participants

A total of 371 Japanese patients with cervical or endometrial cancer who underwent intraoperative sentinel node procedures at Osaka Medical College in Japan between September 2013 and September 2018 were retrospectively reviewed. Patients who met the following criteria were eligible for inclusion in the study: (1) underwent laparoscopic or abdominal hysterectomy with SNB; and (2) underwent an intraoperative frozen section analysis and imprint cytology for the diagnosis of SNB specimens. The present study was approved by the Osaka Medical College Research Review Board, and all patients gave their written informed consent for inclusion in the present study.

### The SLN Mapping Procedure

We previously reported the SNB procedure [1–4]. Briefly, all tracers were sub-mucosally injected in four quadrants of the cervix at 0, 3, 6, and 9 o’clock. A superficial cervical injection to a depth of 3–5 mm below the surface of the cervix was performed in all cases, as described previously [5, 6]. On the day before the operation, 2.0 ml of fluid containing 110 MBq 99 m-Technetium (^99m^Tc)-labeled tin colloids was injected into the patient’s cervix. Lymphoscintigraphy was performed within 6 h, and hot spots (indicating SLNs) were identified. On the day of the operation, 5 ml of indigocarmine (IDC) (2–4 mg/ml) and/or indocyanine green (ICG) (50 μg/ml) was injected into the cervix at the start of surgery. The same quantity of IDC and/or ICG was also injected into the uterine fundus upon reaching the intraabdominal cavity for endometrial cancer. The SLNs were detected 40 min after the injection of IDC or ICG. Radioactive lymph nodes were located using a gamma probe (Navigator GPS, RMD). IDC-stained lymph nodes were detected by direct inspection. ICG fluorescence-positive lymph nodes were detected using a color fluorescence camera (Hyper Wye Medical System, MIZUHO Co., for laparotomy; Camera Control Unit JC300, MC Medical Co., or 1588AIM camera system; Striker Co., for laparoscopy). After SLN biopsy, the area of the pelvic lymph node was surveyed by direct observation, and with a color fluorescence camera or a gamma-probe to confirm that no radioactive tissue remained. The combination of ^99m^Tc and IDC was used in the early phase and a combination of three tracers was used in the late phase.

### Pathological Examinations and the Analysis of SLNs

**Fig. 1:**
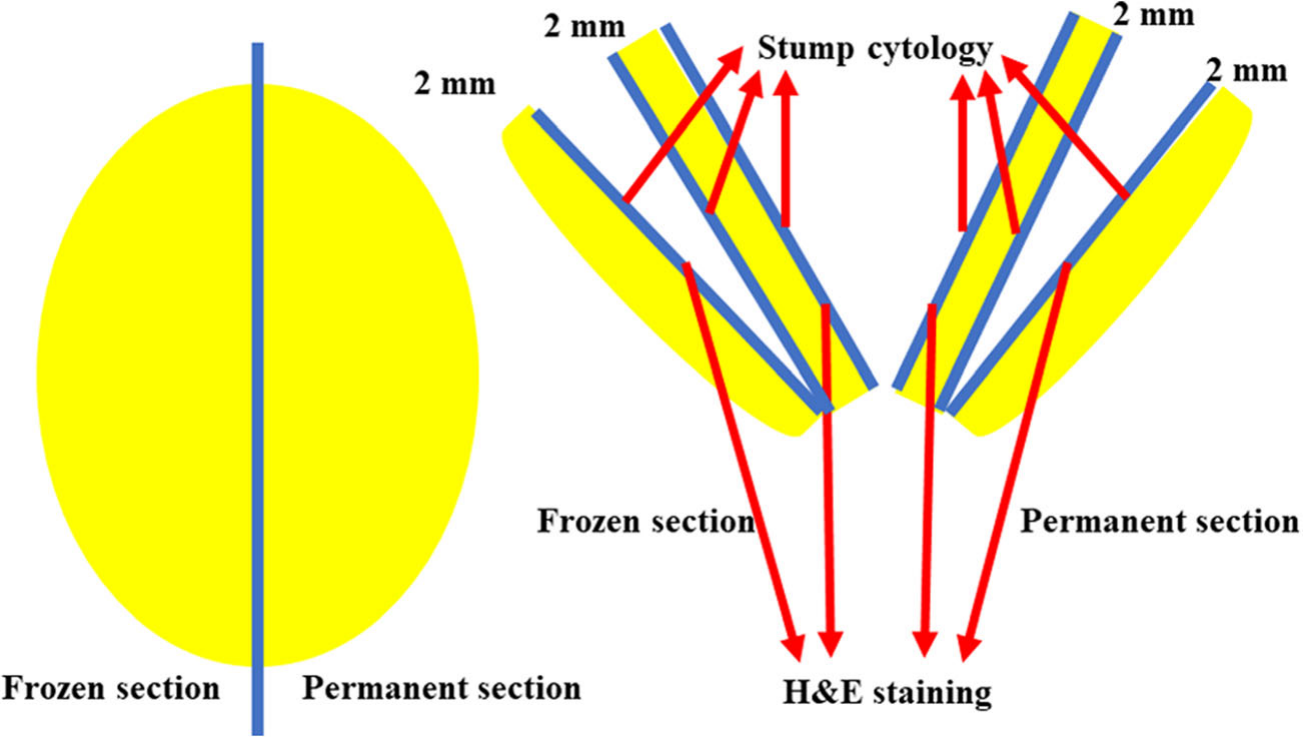
Management of sentinel lymph nodes (SLNs). The SLN was bisected parallel to the longest axis to obtain the maximum section area. One half was used to create a frozen section. The other half was used for a permanent section. The specimen was cut every 2 mm into 5-μm-thick sections, which were stained with hematoxylin and eosin (H&E). When the SLNs were cut, a touch preparation was performed with a glass slide on both sides of the cut

**Fig. 2:**
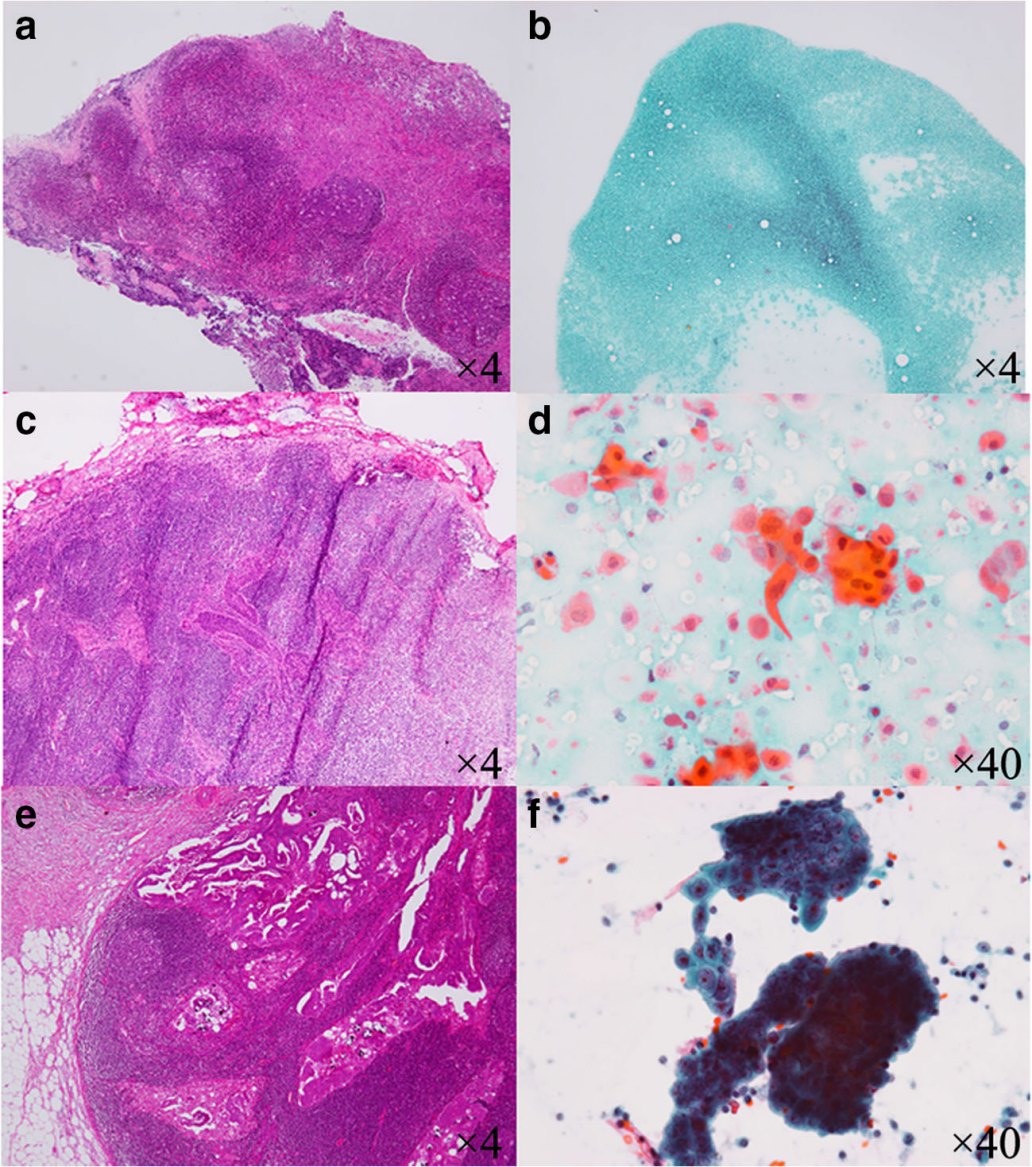
H&E staining of frozen sections (**a**, **c** and **e**) and Papanicolaou staining of imprint cytology (**b**, **d** and **f**) from excised sentinel lymph nodes (SLNs) were evaluated. A and B, frozen section (**a**) and imprint cytology (**b**) of a SLN without metastasis from a patient with endometrial cancer shows a normal population of lymphatic cells. Imaging from imprint cytology shows similar findings to the frozen section. C and D, An SLN with metastasis from a patient with squamous cervical cancer, the irregularly shaped and large nuclei indicate the presence of cancer cells in the frozen section (**c**). On imprint cytology, the cytoplasm of the atypical cells was large and the shape was irregularly round. The cytoplasm is eosinophilic or stained with Orange G (**d**). **e** and **f**, An SLN with metastasis from a patient with serous endometrial cancer. Atypical glands with psammoma bodies are seen in the lymph node (**e**). On imprint cytology, a papillary fragment with an irregular outline is seen. The atypical cells have large nuclei with irregularly distributed chromatin, distinct nucleoli, irregular nuclear margins and an indistinct cytoplasmic border (**f**)

An intraoperative pathological examination was performed (Fig. 1). The SLN was cut in half, parallel to the longest axis, to obtain the maximum section area. One half was used to create a frozen section. The specimen was cut every 2 mm into 5-μm-thick sections, which were stained with hematoxylin and eosin (H&E). The specimens were evaluated when the frozen sections were obtained. The other half of each specimen was also cut every 2 mm and fixed in 10% formalin for a permanent section procedure; the 5-μm-thick sections were stained with H&E and examined. When the SLNs were cut, touch preparation was performed with a glass slide on both sides of the cut. The slide was subjected to Papanicolaou staining and was interpreted by experienced cytologists (Fig. 1). The frozen section analysis of all SLNs was performed as an intraoperative examination by a senior pathologist. In contrast, the diagnosis of imprint cytology was performed postoperatively by two of four cytologists each of whom had >10 years of experience reporting the findings of gynecological cytology. The readers were not aware of the clinical data, including the results of the frozen section analysis and the final pathological diagnosis. In the event that there was disagreement in the readers diagnoses, all members consulted with each other until they reached a consensus (Fig. 2). Frozen tissues were embedded and examined as frozen formalin-fixed and paraffin-embedded tissue. However, there were no cases in which the diagnosis differed between the frozen sections and the formalin-fixed paraffin-embedded tissue sections.

### Sensitivity and Specificity

The sensitivity, specificity and negative predictive value were determined for the frozen section analysis alone, imprint cytology alone, and the combination of the two methods. The frozen-section-positive SLNs were considered positive, even if there were no cancer cells in the other half of the specimen in the final pathological diagnosis. Sensitivity was defined as the rate of positivity by the given assessment and the final pathological diagnosis. Specificity was defined as the rate of negativity by the given assessment and final pathological diagnosis. The negative predictive value was defined as the rate of negativity by the given assessment and the final pathological diagnosis. For the combined diagnosis, an SLN was considered positive if the frozen section was positive, the imprint cytology was positive, or if both analyses were positive.

### Statistical Analyses

All of the statistical analyses were performed using the JMP software program (version. 13.1.0). Continuous variables were expressed as the mean ± standard deviation (SD). The Mann-Whitney U-test was used to compare continuous variables, and Fisher’s exact test was used to compare frequencies. When making multiple comparisons of frequencies in a dataset, Fisher’s exact test with Bonferroni’s correction was used. *P* values of <0.05 were considered to indicate statistical significance.

## Results

Three hundred seventy-one patients with cervical or endometrial cancer underwent SNB with an intraoperative frozen section analysis and imprint cytology. The mean age of the patients was 52.7 ± 12.4 years, and the mean body mass index was 23.2 ± 4.5. Among 146 cervical cancer patients, 22 had International Federation of Gynecology and Obstetrics (FIGO) stage IA disease, 75 had stage IB disease, 26 had Stage IIA disease and 23 had stage IIB disease. According to the histological examinations, 93 patients had squamous cell carcinoma and 53 patients had adenocarcinoma. Among 225 endometrial cancer patients, 194 had FIGO stage I disease, 2 had stage II disease, 25 had stage III disease, and 4 had stage IV disease. According to the histological examinations,144 patients had grade 1 endometrioid carcinoma, 34 had grade 2 disease, 22 had grade 3 disease, 4 had clear cell carcinoma, 15 had serous carcinoma and 6 had carcinosarcoma. Lymph node metastasis was detected in the final pathological diagnosis of 51 of 951 removed SLNs (Table 1). The frozen section analysis and imprint cytology of these specimens did not reveal metastasis in any of the 900 SLNs for which the final pathological diagnosis was negative for metastasis. Among the 51 SLNs with metastasis, 39 were frozen section-positive, 37 were imprint cytology-positive, and 47 were both frozen section- and/or imprint cytology-positive. The sensitivity of the frozen section analysis, imprint cytology, and the combination of frozen section and imprint cytology was 76.5%, 72.6% and 92.2%, respectively. The combination of the frozen section analysis and imprint cytology showed significantly higher sensitivity than the frozen section analysis or imprint cytology alone (*p* = 0.02). The specificity was 100% in each group. The negative predictive values of the frozen section analysis, imprint cytology and the combination of the frozen section analysis and imprint cytology were 98.7%, 98.5% and 99.6%, respectively (Table 2). In cervical cancer, the sensitivity of a frozen section analysis, imprint cytology, and the combination of the frozen section analysis and imprint cytology was 72.7%, 69.7% and 87.9% (*p* = 0.15), respectively. In endometrial cancer, the sensitivity of a frozen section analysis, imprint cytology and the combination of the frozen section analysis and imprint cytology was 83.3%, 77.8% and 100% (*p* = 0.04), respectively. In squamous cell carcinoma, the sensitivity of a frozen section analysis, imprint cytology and the combination of the frozen section analysis and imprint cytology was 70.8%, 75.0% and 91.7% (*p* = 0.14), respectively. In adenocarcinoma, the sensitivity of a frozen section analysis, imprint cytology and the combination of the frozen section analysis and imprint cytology was 81.5%, 70.4% and 92.6% (*p* = 0.10), respectively. These findings suggested that a frozen section analysis tended to show higher sensitivity than other approaches in the diagnosis of adenocarcinoma. Furthermore, imprint cytology improved the sensitivity (Table 3). According to the American Joint Committee on Cancer (AJCC) classification, isolated tumor cells were defined as ≤0.2 mm, micrometastasis was defined as a tumor of >0.2 mm but ≤2 mm and macrometastasis was defined as a tumor of >2 mm [7]. In cases of macrometastasis, the sensitivity of a frozen section analysis, imprint cytology and the combination of the frozen section analysis and imprint cytology was 86.7%, 86.7% and 100% (*p* = 0.03), respectively. In micrometastasis, the sensitivity of a frozen section analysis, imprint cytology and the combination of the frozen section analysis and imprint cytology was 71.4%, 71.4% and 92.9% (*p* = 0.047), respectively. In isolated tumor cells, the sensitivity of a frozen section analysis, imprint cytology and the combination of the frozen section analysis and imprint cytology was 42.9%, 14.3% and 57.1% (*p* = 0.20), respectively (Table 3). Seven SLNs in seven patients were frozen section-negative and imprint cytology-positive. Three patients had endometrial cancer (carcinosarcoma [*n* = 1], clear cell carcinoma [*n* = 1], and grade 1 endometrioid carcinoma [*n* = 1]). The remaining four had cervical squamous cell carcinoma.

**Table 1:** The characteristics of cervical and endometrial cancer patients who received a sentinel lymph node biopsy

Total number of patients	371
Age^a^, years	52.7 ± 12.4
BMI^a^	23.2 ± 4.5
Cervical cancer	146
FIGO stage IA	22
IB	75
IIA	26
IIB	23
Squamous cell carcinoma	93
Adenocarcinoma	53
Endometrial cancer	225
FIGO stage I	194
II	2
III	25
IV	4
Endometrioid G1	144
Endometrioid G2	34
Endometrioid G3	22
Clear cell carcinoma	4
Serous carcinoma	15
Carcinosarcoma	6
Number of SLNs removed	951
Number of metastatic SLNs	51

*BMI* body mass index, ^*99m*^*Tc* 99 m-technetium-labeled tin colloid, *IDC* indigo carmine, *ICG* indocyanine green, *PLND* pelvic lymph node dissection, *PAND* paraaortic lymph node dissection, *PAND* paraaortic lymph node

^a^According to an analysis of variance (mean ± standard deviation)

**Table 2:** The correlation of the results of frozen section analyses, imprint cytology, and combination analyses with the final diagnosis of paraffin-embedded sections of metastatic sentinel lymph nodes

Paraffin section	FS		IC		FS + IC		Total
	No metastasis	Metastasis	No metastasis	Metastasis	No metastasis	Metastasis	
No metastasis	900	0	900	0	900	0	900
Metastasis	12	39	14	37	4	47	51
Total	912	39	914	37	904	47	951
Sensitivity	76.5%		72.6%		92.2%		
Specificity	100%		100%		100%		
NPV	98.7%		98.5%		99.6%		

*FS* rozen section, *IC* imprint cytology, *NPV* negative predictive value

**Table 3:** The sensitivity of frozen section analyses, imprint cytology and combination analyses in the diagnosis of sentinel lymph nodes

	Sensitivity			*p* value
	FS	IC	FS + IC	
	76.5%	72.6%	92.2%	0.02
Disease				
Cervical cancer	72.7%	69.7%	87.9%	0.15
Endometrial cancer	83.3%	77.8%	100%	0.04
Histology				
Squamous cell carcinoma	70.8%	75.0%	91.7%	0.14
Adenocarcinoma	81.5%	70.4%	92.6%	0.10
Tumor size				
Macrometastasis	86.7%	86.7%	100%	0.03
Micrometastasis	71.4%	71.4%	92.9%	0.047
Isolated tumor cells	42.9%	14.3%	57.1%	0.2

*FS* frozen section, *IC* imprint cytology

## Discussion

In the current study, the sensitivity of the frozen section analysis, imprint cytology, and the combination the two modalities in the analysis of SNB specimens was 76.5%, 72.6%, and 92.2%, respectively. The accuracy of the frozen section analysis and imprint cytology in the analysis of SNB specimens was considered acceptable; however, the sensitivity of the combination approach was higher than the sensitivity of frozen section or imprint cytology performed alone.

Pelvic lymph node management in patients with uterine cancer has improved significantly over recent decades, and SNB might become a standard procedure for pelvic lymph node staging in patients without clinically detectable lymph nodes. The National Comprehensive Cancer Network (NCCN) has proposed that pelvic lymphadenectomy should be restricted to certain situations. Intraoperative imprint cytology is comparable to a frozen section analysis in terms of rapidity and reliability, and is associated with reduced cost and better tissue preservation [8, 9]. Imprint cytology of SLNs has been established as an effective modality with high specificity and a wide range of sensitivity rates in breast cancer. In a meta-analysis that included 31 methodologically acceptable surveys, the pooled sensitivity of imprint cytology was 63%, with specificity levels as high as 99%. The pooled sensitivity for the detection of macrometastasis was 81%, while that for micrometastasis was only 22%. The primary tumor size, the prevalence of metastasis and the proportion with evidence of micrometastasis proved to be significantly associated with the patient outcomes in a univariate meta-regression analysis. Moreover, the proportion with evidence of micrometastasis appeared to be the only significant risk factor in a multivariable analysis [10]. In contrast, frozen section analyses had better sensitivity than imprint cytology in three of four studies that directly compared the two modalities [11].

In the literature on SNB for cervical cancer, both imprint cytology and frozen section diagnoses showed high specificity and a wide range of sensitivity. The pooled sensitivity and specificity of imprint cytology was 8.3–56% and 100%, respectively [12–14]. In contrast, the pooled sensitivity of frozen section analyses was 33–100% [12, 15, 16].

There have been few studies about imprint cytology and frozen section analyses of SNB specimens for endometrial cancer. Ballester et al. compared the diagnostic accuracy of frozen section analyses and imprint cytology in the intraoperative examination of SLNs in early-stage endometrial cancer. The pooled sensitivity and specificity of imprint cytology was 33% and 100%, respectively. In contrast, the pooled sensitivity of frozen section analyses was 86% [17]. The sensitivity of imprint cytology was lower than that in the present study. In their study, each SLN was sectioned at 3-mm intervals, which were analyzed at four additional levels of 200 μm and 4 parallel sections. One was used for H&E staining, and H&E-negative sections were subjected to an immunohistochemical analysis with an anticytokeratin antibody cocktail. Furthermore, most false-negative cases involved micrometastasis or isolated tumor cells on imprint cytology. Their results indicate that it is difficult to detect small cancer cells on imprint cytology. The results were likely similar in our study because we performed frozen section analyses and imprint cytology for all SLNs. In addition, we did not perform ultra-staging. Thus, there is a possibility that we only detected cancer cells near the cut surface.

Frozen section analyses tend to have higher sensitivity than imprint cytology in SNB for the intraoperative diagnosis of patients with cervical and endometrial cancer. In contrast, imprint cytology displays varied sensitivity, however, the specificity and negative predictive value are high. A range of factors may influence this varied sensitivity, including the method of SLN slide preparation, tumor size, and the lymphovascular involvement [18, 19]. It has also been reported that slicing at 2-mm intervals along the short axis could be effective for increasing the accuracy of intraoperative SLN examinations [20, 21].

Endosalpingiosis is defined as the presence of tubal-like epithelium outside of the fallopian tube. In breast cancer, endosalpingiosis is rarely seen in axillary sentinel nodes; however, it may be misinterpreted as metastasis of adenocarcinoma [22]. Endosalpingiosis may be found in pelvic lymph nodes. The reported incidence was 3–4% in uterine or ovarian cancer patients [23, 24]; however, the rate according to the number of lymph nodes or SLNs is uncertain. In the present study, no endosalpingiosis was found in any frozen sections; however, two patients had endosalpingiosis permanent sections of two sentinel nodes. These two cases of endosalpingiosis were in sentinel-negative permanent sections.

The present study was associated with some major limitations, which may reduce its value. First, a significant difference may not have been achieved because of the low rate of nodal metastasis. Second, a multivariate analysis was not performed because of the relatively small sample size. Third, we only used one half of the sentinel nodes to create frozen sections. Fourth, immunohistochemistry with ultra-staging was not performed. As such, our results must be confirmed in further investigations.

In conclusion, both frozen section analyses and imprint cytology displayed acceptable accuracy in the diagnosis of SNB specimens, However, the combination approach showed higher sensitivity in comparison to a frozen section analysis or imprint cytology alone.
